# PeptideVisualizer: A Novel Software Solution for PROTOMAP Analysis

**DOI:** 10.1021/acs.jproteome.5c01209

**Published:** 2026-04-15

**Authors:** Matej Kolarič, Sara Ivanovski, Tilen Sever, Boris Turk, Marko Fonović

**Affiliations:** † 61790 Jožef Stefan International Postgraduate School, Jamova 39, SI-1000 Ljubljana, Slovenia; ‡ Department of Biochemistry, Molecular and Structural Biology, Jožef Stefan Institute, Jamova 39, SI-1000 Ljubljana, Slovenia; § Faculty of Chemistry and Chemical Technology, University of Ljubljana, Večna pot 113, SI-1000 Ljubljana, Slovenia

**Keywords:** PeptideVisualizer, mass spectrometry, proteolysis, PROTOMAP, open-source software, MaxQuant

## Abstract

PeptideVisualizer is an open-source software for PROTOMAP analysis, offering an intuitive graphical user interface and command-line compatibility. The software processes mass spectrometry data output from MaxQuant to visualize protein migration in polyacrylamide gel electrophoresis and sequence coverage. In addition, it integrates information regarding protein secondary structure and features from the UniProt database to visualize them within comprehensive peptographs. These features allow us to assess the occurrence of a proteolytic event based on sequence context and peptides abundance. The key advantages of PeptideVisualizer include the integration of quantitative information, handling of multiple experiments with biological replicates, and the introduction of a novel mismatch factor—a metric designed to rapidly identify proteolytic events. Herein, the software was validated in an apoptosis-related data set, demonstrating its effectiveness and usefulness in large-scale proteomic data analysis.

## Graphical abstract

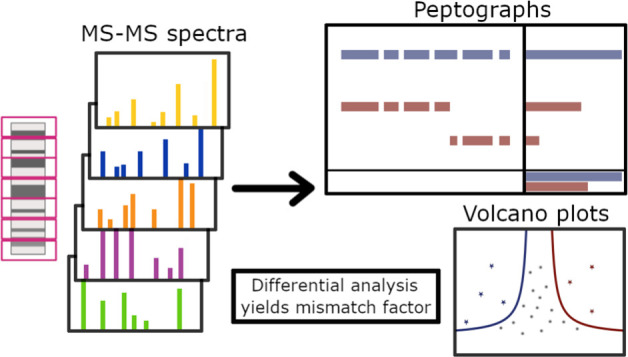

## Introduction

Proteases are present in all living cells and cleave proteins and peptides by hydrolyzing peptide bonds. This hydrolysis can lead to the total degradation of a protein, such as in food digestion and cellular protein recycling, or a highly specific structural modification that affects its function. Through this irreversible modification, proteases regulate numerous physiological processes in health and pathology, including cell cycle regulation, immune response, wound healing, and cancer, as well as cardiovascular, neurodegenerative, and infectious diseases.^1^ In the latter, the invaders often utilize their proteases or host proteases to facilitate pathogenicity, such as during SARS-CoV-2 infection.^2,3^ Thus, several drugs have been developed to target proteases, with many of them in various stages of clinical trials.^4–7^ Therefore, understanding protease activity is of immense significance in the advancement of novel therapeutics.^8^ The identification of proteases, their substrates, and their inhibitors on a system-wide scale is termed degradomics. Various approaches are used in this field, with mass spectrometry-based proteomics being the gold standard.^9,10^

Mass-spectrometry-based proteomics can be categorized into protein-centric and peptide-centric approaches. Protein-centric approaches investigate intact proteins and their fragmentation products. Although this method is suitable for smaller proteins, their ionization, detection, and quantification become challenging as the size increases. Hence, peptide-centric approaches in which proteins are initially digested into peptides and these are further analyzed to obtain information about the parent protein are the method of choice for most mass spectrometry-based proteomic approaches. This technique overcomes the problems associated with the analysis of not only large proteins but also membrane proteins, low-abundant proteins, and those with aberrant sequence modifications.^11^ PROTOMAP (PROtein TOpography and Migration Analysis Platform) is a peptide-centric approach in which mass-based fractionation of proteins on polyacrylamide gel is utilized to reduce sample complexity. Proteins from each slice are separately digested and analyzed using mass spectrometry to determine sequence coverage and to quantify those in any given fraction. Similar to immunodetection coupled to Western blotting, this method enables the detection of a mass change. However, instead of being performed for a single protein, it is conducted quantitatively for all detected proteins, with the resolution determined by the number of fractions. The method was developed in 2008, when the Cravatt group provided software for data analysis, described its typical workflow (Figure 1), and demonstrated its successful usage.^12^ The software solution comprises three steps that utilize a SEQUEST search algorithm running on MS2 files, the DTAselect 2.0 tool to filter the obtained peptides, and finally, three Perl scripts to create output images and a result file.

**Figure 1:**
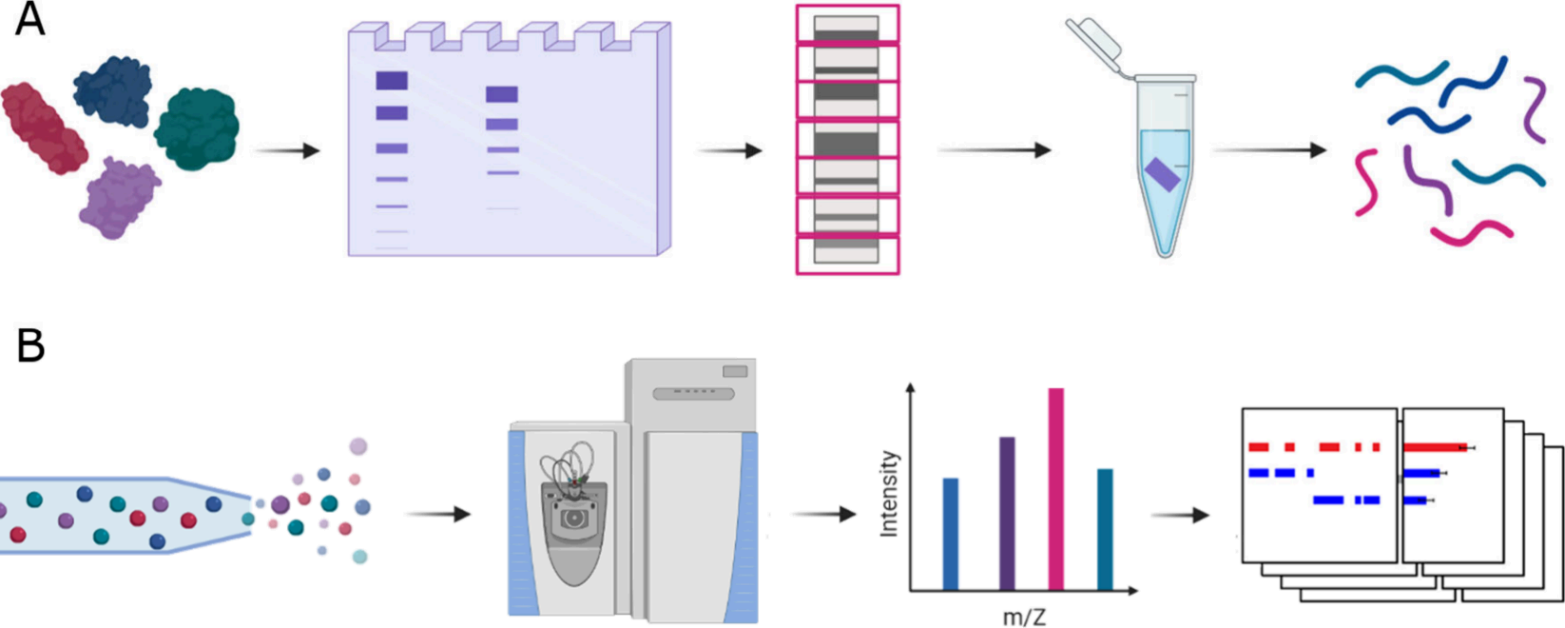
Typical PROTOMAP workflow. (A) Sample preparation. A complex protein sample is separated using SDS-PAGE, and the resulting gel lanes are partitioned into fractions. Each fraction is then in-gel prepared for tandem mass spectrometry. (B) LC-MS/MS and bioinformatic analysis. Peptides are separated by LC and ionized using ESI for mass spectrometry. Acquired *m*/*z* spectra are used for peptide identification and quantification. The results are processed to generate peptographs.

This method was improved using Pep2Graph, another software solution for analyzing PROTOMAP data.^13^ Pep2graph features three key improvements, allowing the comparison of up to six conditions, and is ready to use with the output of the MaxQuant software.^13^ Although Pep2Graph presented the community with some improvements, several interesting features remained unaddressed, including support for multiple replicates and the analysis of quantitative data. Furthermore, to detect proteolytically processed proteins, a peptograph for each detected protein had to be manually checked and interpreted, which is a time-consuming and labor-intensive process. Addressing the latter issue is crucial to unlock the complete potential of the PROTOMAP method.

## Materials and Methods

### Cell Culture and Preparation of Whole-Cell Lysates

Jurkat cells were grown to confluence in RPMI-1640 media supplemented with 10% FBS, 1% penicillin/streptomycin, and 1% glutamine (all Sigma-Aldrich) in a humidified incubator at 37 °C and 5% CO_2_. Eight million cells per condition were treated with Fas ligand (Sigma-Aldrich) to a final concentration of 0.5 μg/mL. Cells were centrifuged after 16 h of treatment, washed twice with phosphate-buffered saline, and resuspended in lysis buffer (50 mM Tris/HCl, 150 mM NaCl, 0.1% SDS, 1% Triton-X-100, 0.5% sodium deoxycholate, and 1% protease inhibitor cocktail (Sigma-Aldrich), pH = 8.0). The cell lysates were centrifuged at 14,000 × g for 5 min at 4 °C. The concentration of proteins was determined with the Bradford assay. Proteins were denatured for 5 min at 95 °C, together with loading buffer, and stored at −20 °C for further use.

### Mass Spectrometry Sample Preparation

The lysate was collected and loaded onto an 8%–16% Tris-Glycine Gel (Invitrogen). After separation, the gel was stained with Coomassie Brilliant Blue and destained overnight using a solution of 30% ethanol and 10% acetic acid. The gel was rehydrated with 25 mM ammonium bicarbonate for 1 h, followed by reduction with 10 mM DTT at 37 °C. Free sulfur was alkylated with 55 mM iodoacetamide under basic conditions for 30 min in the dark. Subsequently, the gel was rinsed with 25 mM ammonium bicarbonate for 15 min and cut into 30 pieces of the same size. Two neighboring pieces of gel bands were put together in a fresh tube. In the next step, the gel pieces were destained overnight at 4 °C with 25 mM ammonium bicarbonate in 50% acetonitrile, followed by a 15 min wash with acetonitrile only. Afterward, the gel pieces were vacuum-dried before trypsinization with approximately 1 μg of sequencing-grade porcine trypsin (Promega) in 25 mM ammonium bicarbonate at 37 °C overnight. Peptides were extracted with 0.1% formic acid at 1,200 rpm and later desalted using three stacks of C18 disks (Empore) in a 200-μL pipet tip. The C18 stacks were conditioned in the following order: 100% methanol, 0.1% formic acid in acetonitrile, and 0.1% formic acid. The peptides were eluted with 0.1% formic acid in 60% acetonitrile. For liquid chromatography-tandem mass spectrometry (LC-MS/MS) analysis, the samples were further concentrated to a final volume of 15 μL.

### LC-MS/MS Analysis

LC-MS/MS analysis was performed using an EASY nanoLC II high-performance liquid chromatography unit (Thermo Scientific) coupled to an Orbitrap LTQ Velos mass spectrometer (Thermo Scientific) with the Xcalibur software (Thermo Scientific). The peptide samples were loaded on a C18 trapping column (Proxeon EASY-Column, Thermo Scientific) and separated on a C18 PicoFrit Aquasil analytical column (New Objective). The peptides were eluted with a 90 min linear gradient using 5%–50% acetonitrile and 0.1% formic acid at a flow rate of 300 nL/min. The complete MS spectra were acquired at a resolution of 30,000 with a mass range of 300–2,000 *m*/*z* using an Orbitrap mass analyzer. The MS/MS spectra were obtained for the nine most intense MS precursor ions recorded at a resolution of 7,500, which were fragmented using HCD fragmentation. For MS/MS fragmentation, only precursor ions with assigned charge states (>1) were used. The dynamic exclusion was set to a repeat count of 1, a repeat duration of 30 s, and an exclusion duration of 20 s.

### Data Analysis

MaxQuant proteomics software (version 2.1.4.0), equipped with an embedded Andromeda search engine, was used to perform database searches against the *Homo sapiens* UniProt database (UniProtKB, 20,408 entries, released July 2023), utilizing trypsin cleavage specificity with a maximum of three missed cleavages. Carbamidomethylation of cysteines was set as a fixed modification, whereas methionine oxidation and N-terminal acetylation were set as variable modifications. For peptides and proteins, a database search was performed with an identification-reversed approach, using a false discovery rate (FDR) of 1%. Each sample was considered as an individual group to allow PROTOMAP analysis.

The PROTOMAP analysis was performed using PeptideVisualizer (version 1.9f). The experiments were conducted in two separate groups: control (C) and experimental (F). The replicate number was set to three. The LFQ intensities were enabled, the posterior error probability (PEP) filter was set to be <5%, and the peptide threshold was set to be <5%. The UniProt online database was used, with visualization of all features enabled. FDR was disabled, and the fudge factor s_0_ was set to 0.2.

## Results and Discussion

Here, we present PeptideVisualizer, a free, open-source, cross-platform, and easy-to-use Python 3 software solution for the PROTOMAP analysis. The software performs analysis on the output generated by the free and widely used MaxQuant software. While other proteomics search engines, such as MSFragger are increasingly adopted by the community, direct support for these platforms is not yet implemented. To address a dedicated parsing function will need to be developed to work with different result files.

PeptideVisualizer features a simple-to-use graphical user interface (GUI) while maintaining backward compatibility with the command-line interface (CLI), which is used automatically only if the operating system does not support a GUI. The major improvements include the quantitative visualization of mass spectrometry data, integration of protein secondary structure information, support for comparing multiple conditions with several replicates featuring replicate quality control, imputation of missing data, and detection of significantly dysregulated proteins. Furthermore, we present a novel mismatch factor that provides the user with a parameter reflecting the difference between two experimental conditions, enabling the rapid detection of proteolytic events. Hence, each result need not be reviewed manually. Finally, we developed an intuitive result viewer to quickly check numerous proteins, bookmark interesting proteins, and export saved selections. The PeptideVisualizer is available for download at www.proteome.eu and https://github.com/kolocode/Peptide-Visualizer where additional instructions on configuring MaxQuant and using PeptideVisualizer can be found.

When PeptideVisualizer is opened, the CLI begins running, providing the researcher with progress updates, potential warnings, and error output (Figure S1). The software initially checks if all the required dependencies are installed and attempts to install the missing ones. Subsequently, it performs various other checks, such as online connectivity, GUI compatibility, and the availability of a new version. After all the checks are completed and the requirements (i.e., Python dependencies) are installed, the user is prompted to provide the peptides.txt file output generated by the MaxQuant software. The experimental data are detected from the provided file, and the main GUI is opened (Figure S2). The user then groups the detected experiments as desired, while various analysis settings and parameters can be set. The main GUI is equipped with tooltips, allowing the user to obtain descriptions and instructions about any feature by simply hovering the mouse over an element. The number of replicates must be specified by the researcher, and the remaining options can retain their default settings. When the “Submit Experiment” button is pressed, a second window opens, displaying an example peptograph. Here, the user can annotate the vertical axis and select combinations of interest, which will be considered for calculating the mismatch factor and generating volcano plots to detect dysregulated proteins (Figure S3).

The progress, comprising four major steps, is shown in the CLI until the analysis is complete. **(I)** In the first step, the FASTA sequences and protein features are retrieved from UniProt if “Use UniProt online DB” is selected from the options and the computer is connected to the Internet. Otherwise, the user is asked to provide a FASTA file, which was used to generate the MaxQuant result; the entire analysis can then be performed offline. If the “Use UniProt online DB” option is selected, additional options to download and display protein features, including proteolytic processing, protein regions, and secondary structures, become available. The first step is complete when the peptides.txt result file from MaxQuant, protein sequences, and protein features are loaded into the computer’s working memory. **(II)** In the second step, peptide filtering and statistical analysis are carried out. Peptides are first filtered according to the PEP filter. Peptide intensities are then normalized to construct matrices, which are used for the generation of the final results. Normalization is performed by rescaling (min–max normalization), where the minimum is set to 0, hence each value is divided by the maximum peptide intensity of a given protein. Total protein intensities for each condition are subsequently obtained by summing the intensities of all peptides assigned to that condition. Furthermore, protein sequence coverages are created in text and HTML formats, which are integrated into the final results, allowing the user to see precisely which peptides were detected in each condition. Later, a mismatch factor is calculated for each combination of interest to help the user quickly detect proteolytic events and other variables, such as sorting lists and summaries, which facilitate the generation of the final results display. The mismatch factor is calculated as a sum of values for each peptide in the protein; if a peptide is detected in the same fraction, a negative peptide value is assigned, and if a peptide is detected in another fraction, a positive peptide value is assigned. This value is multiplied by the distance between fractions. Hence, the value becomes low if the same peptides are in the same fraction or in a nearby fraction, whereas the number becomes high if the same peptide is detected in another fraction, and the value is proportional to the separation between fractions (Equation S1). Therefore, the mismatch factor reflects the proteolytic activity. **(III)** The third step is only performed if multiple replicates are provided. This step comprises correlation analysis of quantified proteins between replicates for quality control. Missing values are subsequently imputed using two different approaches: multiple imputation by chained equations (MICE) and imputation from a Gaussian distribution with a median down-shift of 1.8 and a distribution width of 0.5 relative to the quantified proteins, simulating the assumption that missing values arise from low-abundance proteins.^14,15^ Volcano plots are then generated for each imputation type and combination of interest to identify dysregulated proteins. These proteins are then used to draw protein networks and retrieve GO enrichment data via the STRING application programming interface.^16^ After the third step, the HTML result file integrating previously generated files is created and opened in the researchers’ default web browser (Figure S3). These steps are completed in a few minutes. **(IV)** The final step is the actual drawing of peptographs, which may require several hours, depending on the number of detected proteins and the availability of hardware.

A crucial improvement in peptograph generation is the visualization of quantitative information in the form of intensity or LFQ intensity, depending on the settings. The quantitative information is displayed for each peptide, with the opacity of the rectangle representing the relative intensity of the peptide. In addition, in the right quarter of the peptograph in which MS-count information is conventionally displayed, the gross slice intensity or LFQ intensity for a given condition is exhibited using a horizontal histogram. Another key enhancement is the visualization of protein feature information, namely, molecular processing, regions, and secondary structure. These details are presented in the three lines at the bottom of the peptograph, as depicted in Figure 2.

**Figure 2:**
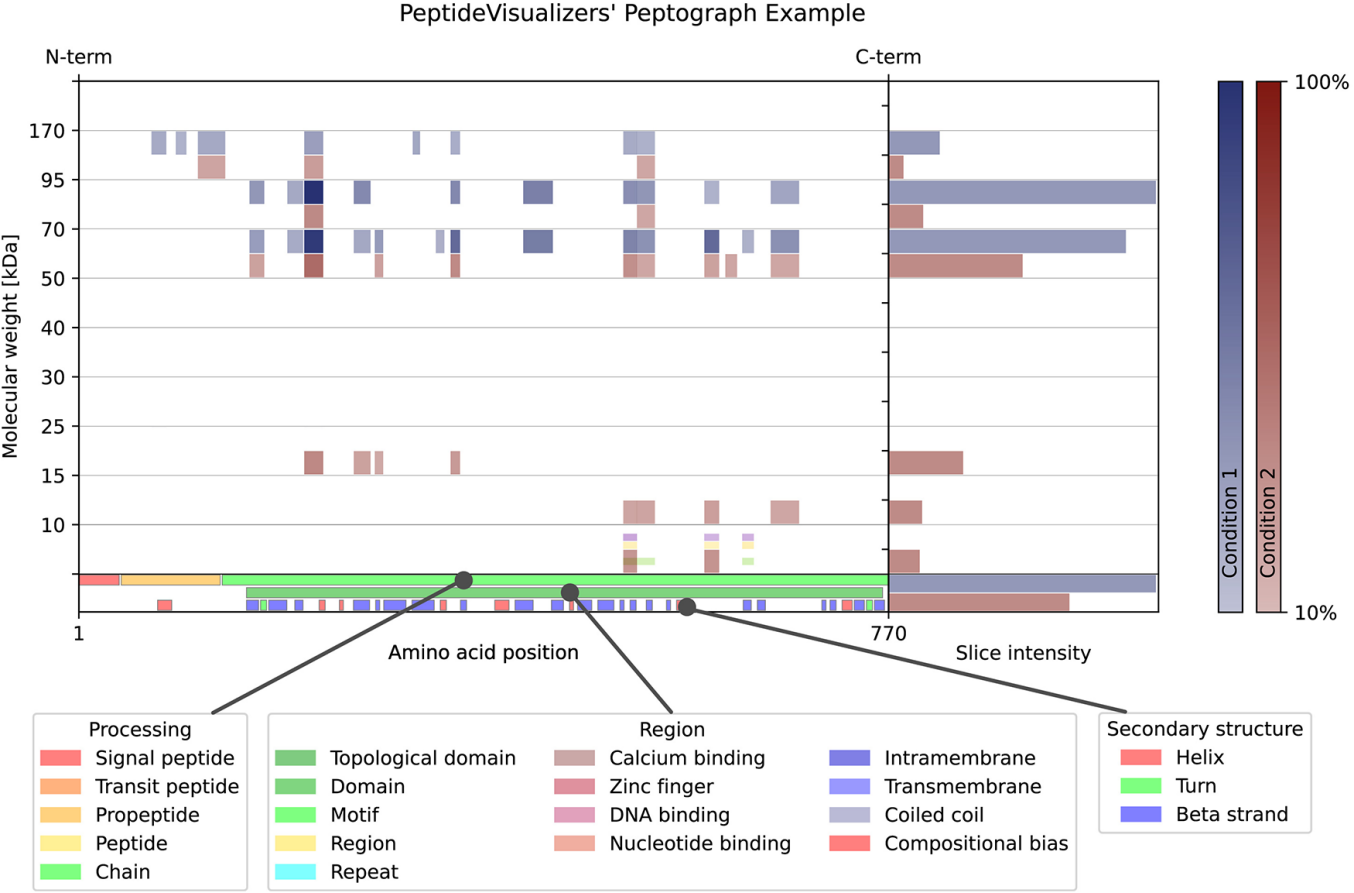
An example of a peptograph from the novel PeptideVisualizer software solution, with a legend for protein feature visualization. Peptides from two different conditions are colored in blue and red.

The HTML result file comprises three sections. When opened, the main selection contains instructions for using the result file, a visual representation of the experimental grouping, a feature visualization legend, and command-line output information, encompassing all the settings used and required to reproduce the analysis. This page can be accessed by pressing the “H” key or clicking on the homepage icon (Figure S5). In case of multiple replicates, the home page also provides the user with a menu to show replicate correlations (Figure S6), imputation visualization (Figure S7), and volcano plots (Figure S8). These options are generated for each of the previously selected combinations of interest, together with gene lists of proteins that are upregulated or downregulated in a statistically significant manner. The list of all the detected proteins is displayed at the top. Several parameters are provided in the list, including the mismatch factor, sequence coverage, peptide count, and the second logarithm of the protein gross intensity. The protein list can be sorted according to any of the provided parameters by clicking on the desired parameter. The results for a specific protein, containing the peptograph and the sequence coverage, can be accessed by clicking on its name in the protein list. The next or the previous protein, according to the selected sorting factor, can be quickly accessed by pressing the right or left arrow key, respectively. The bookmark section is displayed to the right of the result file. Whenever the user classifies a protein as relevant, it can be quickly added to the bookmark selection by pressing the “S” key or removed by pressing the “R” key. This feature provides the user with “hands on the keyboard” ability and does not require mouse usage to quickly review the results. Together with a novel mismatch factor that substantially reduces the number of peptographs requiring manual checking, this feature provides the opportunity to rapidly review all the results.

Existing tools for PROTOMAP analyses remain limited (Table 1). The original PROTOMAP implementation relies on the user to create complex configuration files for each of multiple processing steps and requires the user to check the peptograph of each protein. Pep2Graph introduced a graphical user interface to make it easier to use, but does not support multiple replicates, cross-platform support, and necessitates manual inspection of peptographs for either targeted proteins or the entire detected proteome to identify proteolytic events. In this regard, the PeptideVisualizer calculates a mismatch factor for each protein, allowing users to prioritize only high-scoring candidates for manual verification.

**Table 1:** Comparison of Available Software for PROTOMAP Analysis

	Initial PROTOMAP	Pep2Graph	PeptideVisualizer
MaxQuant output	X	**√**	√
Replicates support	√	X	√
Quantitative information	X	X	√
Structural information	X	X	√
Cross-platform support	√	X	√
Mismatch factor^a^	X	X	√

a The key feature for the quick detection of proteolytic events.

Our proof-of-concept experiment aimed to compare PeptideVisualizers’ results with those in the original PROTOMAP paper in a similar setting. In the original paper,^17^ the PROTOMAP analysis was performed on data gathered from Jurkat T cells treated with the pan-kinase inhibitor staurosporine to induce the intrinsic apoptosis pathway. Our data were obtained from the same cell line treated with the Fas ligand to induce the extrinsic apoptosis pathway. By analyzing the data and cross-referencing them to the original PROTOMAP data, we identified several proteolytic events common to the intrinsic and extrinsic apoptotic pathways. Notably, poly [ADP-ribose] polymerase one (PARP1) was detected in apoptosis, similar to what was described earlier^12^ (Figure 3).

**Figure 3:**
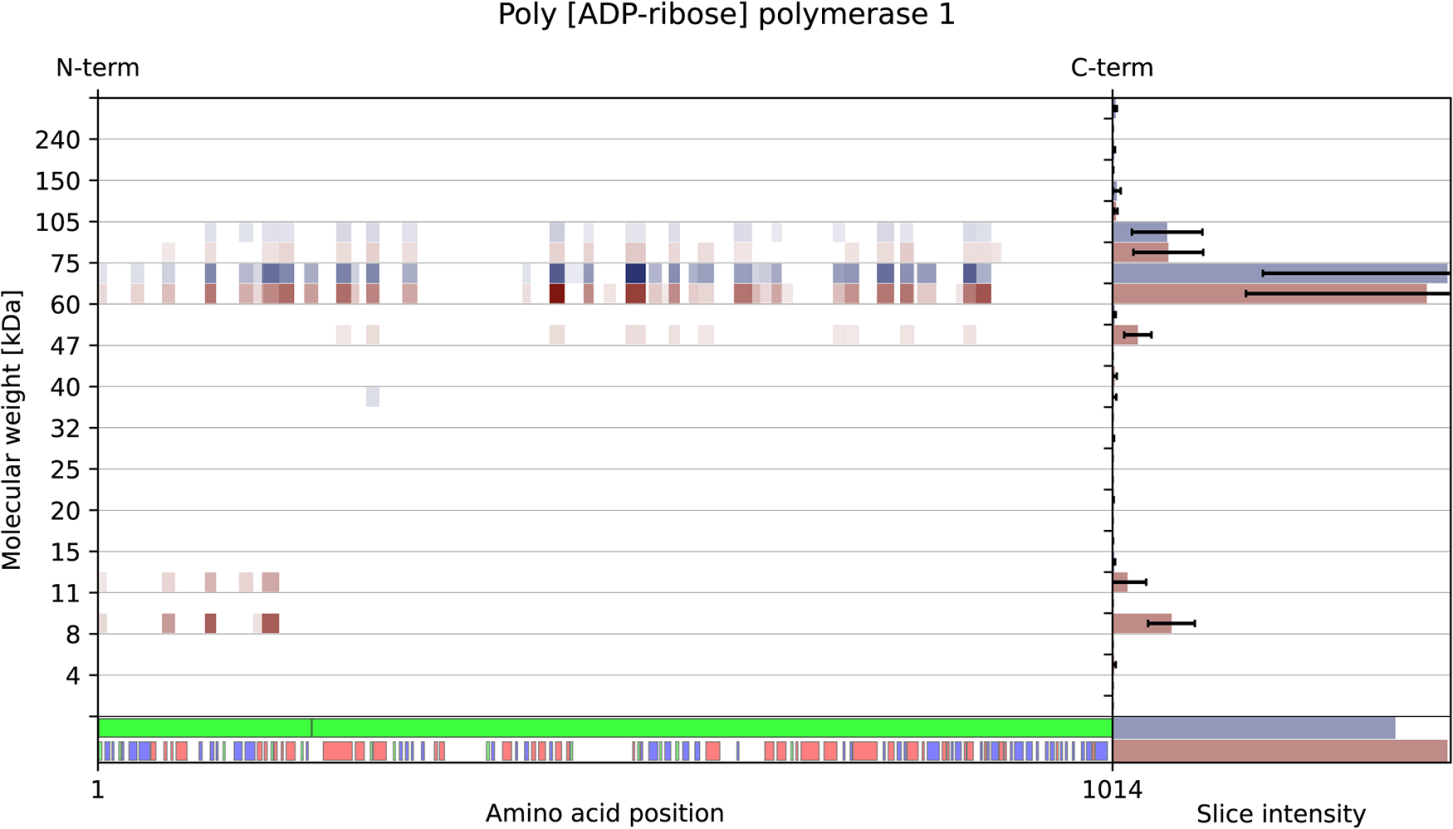
Peptographs of PARP1 obtained using the PeptideVisualizer. The control condition is shown in blue, and the apoptotic condition is shown in red. The error bars represent standard deviation.

Furthermore, our data were compared with those from other studies examining apoptosis. We confirmed the presence of cleaved laminin B1, a well-known marker of apoptosis (Figure 4a).^18^ In addition, we identified nucleolin, known to play a role in RNA processing and known to be cleaved during apoptosis (Figure 4b).^19^ Furthermore, our study uncovered several other substrates that were previously unknown to be cleaved during apoptosis, namely, coatomer subunit delta (Figure 4c) and cleavage and polyadenylation specificity factor subunit 7 (Figure 4d). These findings shed new light on potential proteolytic targets during the progression of apoptosis.

**Figure 4:**
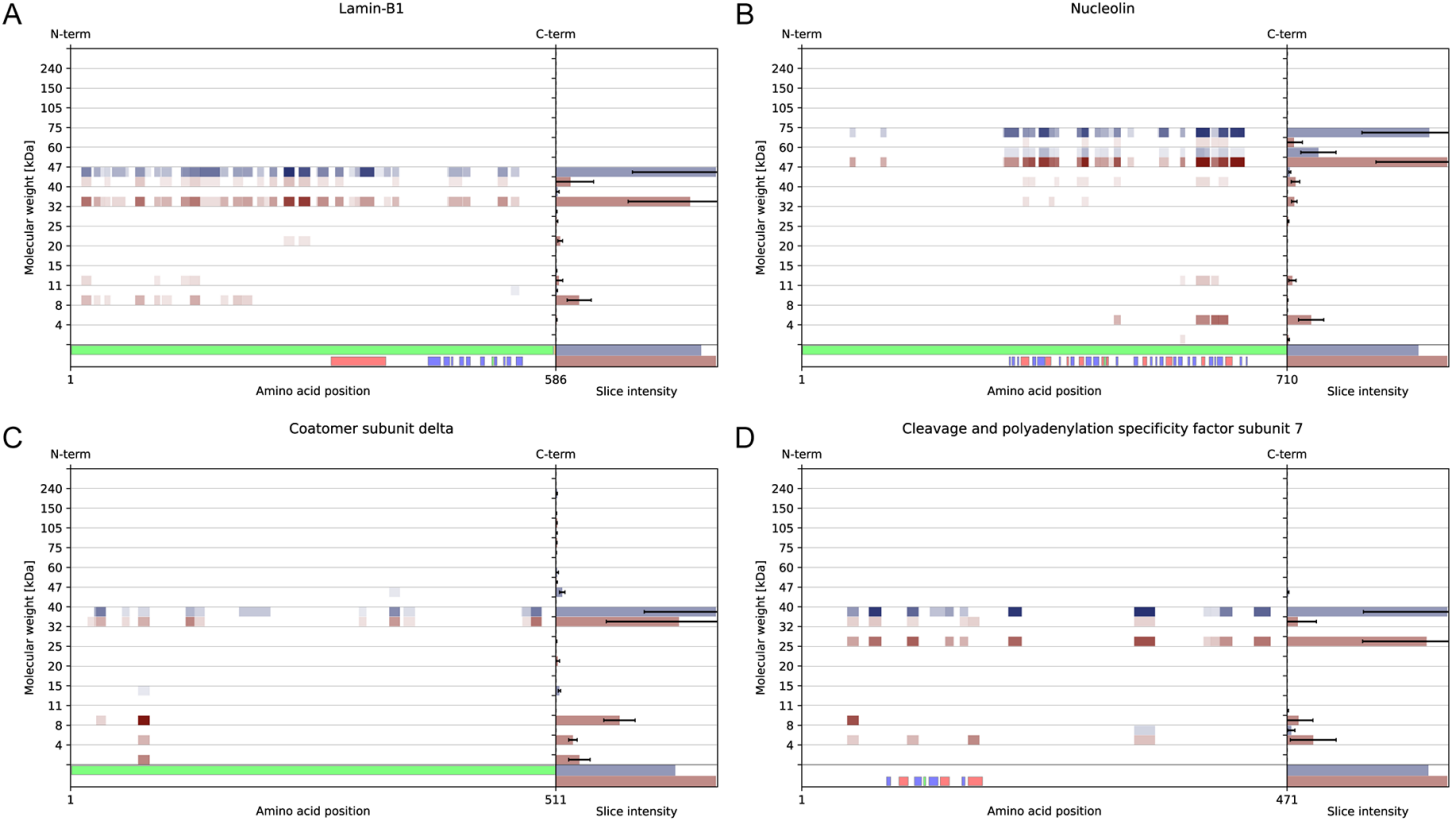
Peptographs obtained using the PeptideVisualizer. The control condition is shown in blue, and the apoptotic condition is shown in red. The error bars represent standard deviation.However, our study demonstrated that all these potentially relevant proteolytic events could be detected using less labor time. All the proteins shown exhibited a novel mismatch factor value in the top 5% of the distribution. In addition, the bottom 80% of proteins by a mismatch factor had no proteolytic relevance. Hence, only 10%–20% of the detected proteins must be checked by the researcher. This finding provides further evidence that our novel mismatch factor will significantly reduce the researcher’s workload.

Building on previous work, our proof-of-concept study validates existing knowledge. Furthermore, PeptideVisualizer offers the research community novel parameters, allowing to focus on proteins that require manual review and discarding those without proteolytic relevance.

## Supplementary Material



## Data Availability

Raw proteomics data generated during this work was deposited to the ProteomeXchange Consortium with PRIDE and is available under identifier PXD071266.

## Notes

Figure S1. Command-line interface output. Figure S2. Main graphical user interface. Figure S3. Example peptograph. Figure S4. Visual schematic mapping of the data flow. Figure S5. HTML results file. Figure S6. Replicates quality control diagram. Figure S7. Imputation graphs example. Figure S8. Volcano plot example. Equation S1. Mismatch factor equation (PDF)

## Abbreviations

CLI: Command-line interface;
FDR: False discovery rate;
GUI: Graphical user interface;
PROTOMAP: PROtein TOpography and Migration Analysis Platform.
