# Supplementary material for: PeptideVisualizer: A Novel Software Solution for PROTOMAP Analysis
Source: J Proteome Res. 2026 Apr 15;25(5):2591–7. doi: 10.1021/acs.jproteome.5c01209 (PMC13140598; doi:10.1021/acs.jproteome.5c01209)
Supplement: Supplementary file 1 [file pr5c01209_si_001.pdf]

PeptideVisualizer: a novel software solution for PROTOMAP analysis

AUTHORS: *Matej Kolarič*[1,2], *Sara Ivanovski*[1,2], *Tilen Sever*[1,2], *Boris Turk*[1,2,3], *Marko Fonović*[1,2,*]

* marko.fonovic@ijs.si, +386-477-3474

[1] Jožef Stefan International Postgraduate School, Jamova 39, SI-1000 Ljubljana, Slovenia

[2] Jožef Stefan Institute, Department of Biochemistry, Molecular and Structural Biology, Jamova 39, SI-1000 Ljubljana, Slovenia

[3] University of Ljubljana, Faculty of Chemistry and Chemical Technology, Večna pot 113, SI-1000 Ljubljana, Slovenia

Table of contents

- Figure S1. Command-line interface output
- Figure S2. Main graphical user interface
- Figure S3. Example peptograph
- Figure S4. Visual schematic mapping of the data flow
- Figure S5. HTML results file
- Figure S6. Replicates quality control diagram
- Figure S7. Imputation graphs example
- Figure S8. Volcano plot example
- Equation S1. Mismatch factor equation

SUPPORTING INFORMATION

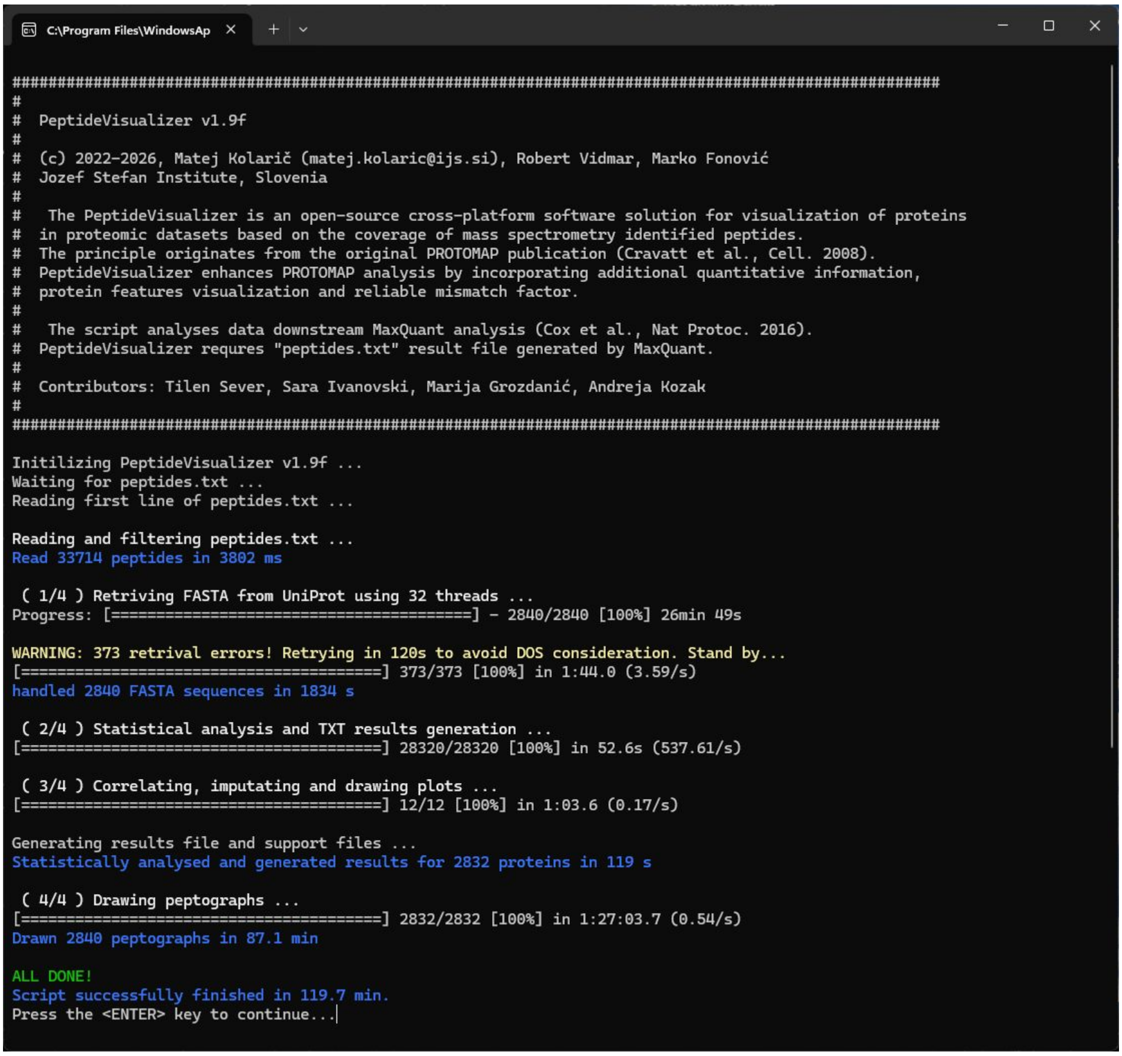

Figure S1: Command-line interface output. The blue text lines separate different processing steps and provide the user with elapsed time information. The green text indicates successful completion, the yellow line indicates a warning, and a red line would indicate error.

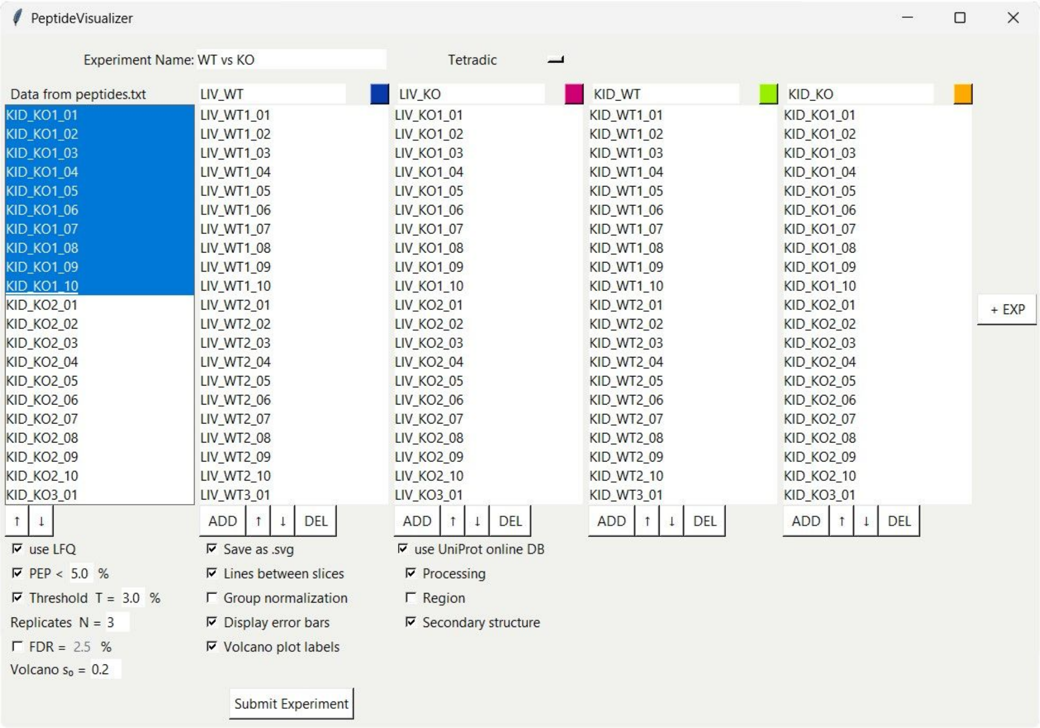

Figure S2: Main graphical user interface. The first column lists all the detected experiments while other are used to group conditions, set their name and color. At the bottom several analysis and/or visual options are available to the researcher.

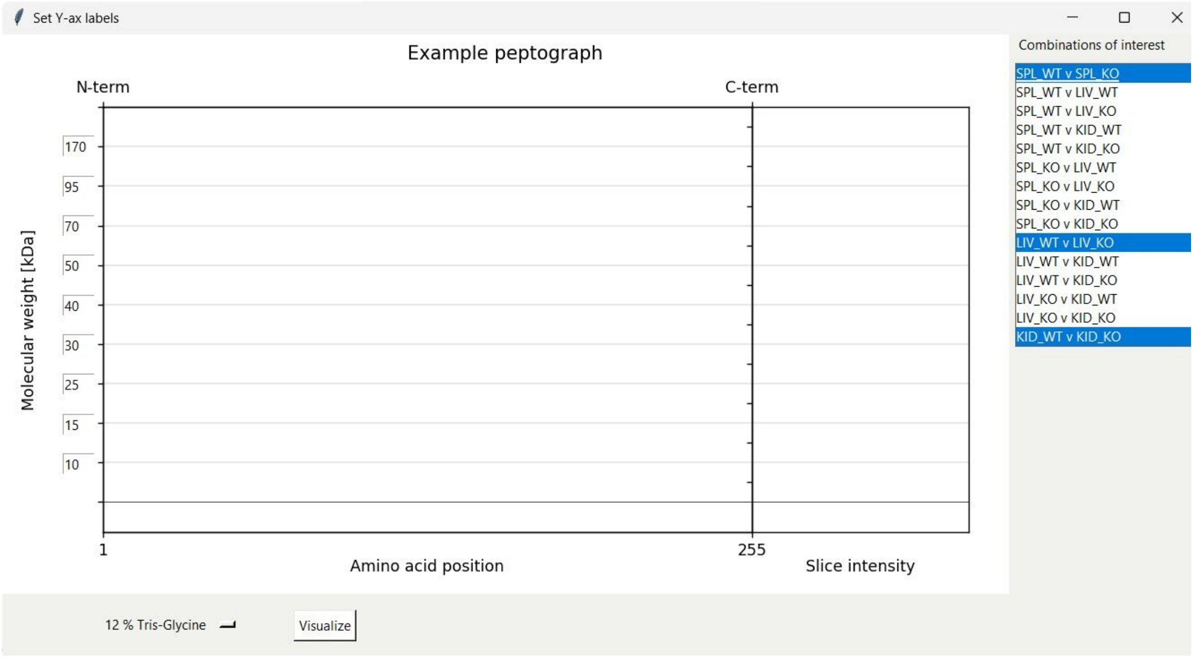

Figure S3: Example peptograph with selected combinations of interests and SDS-PAGE gel preset

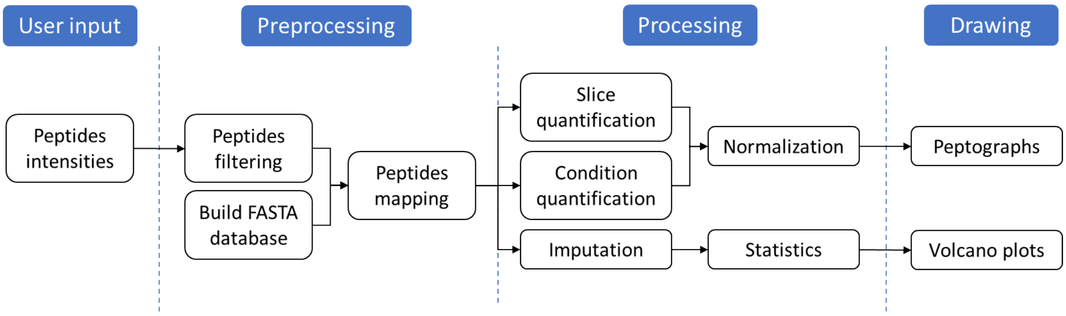

Figure S4: Visual schematic mapping of the data flow

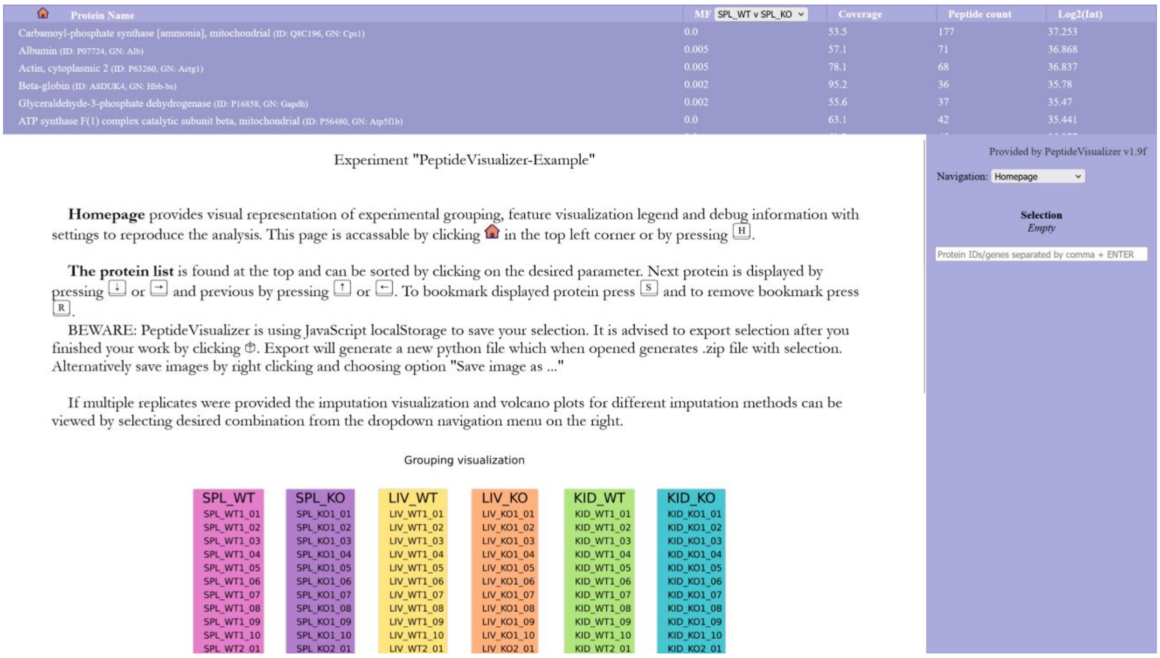

Figure S5: HTML results file. Six different conditions are shown in various colors.

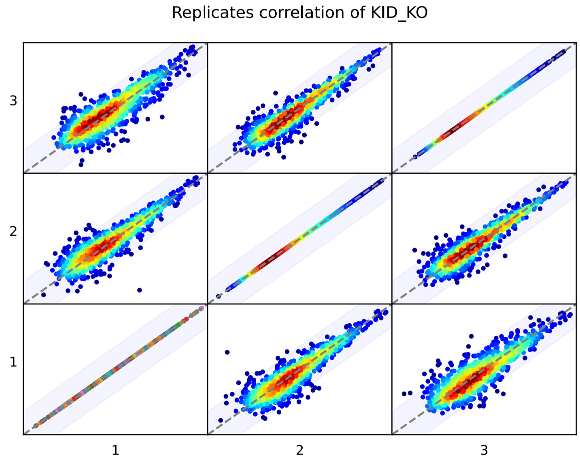

Figure S6: Replicates quality control diagram for a single condition. The warmer areas of the diagram indicate higher data density

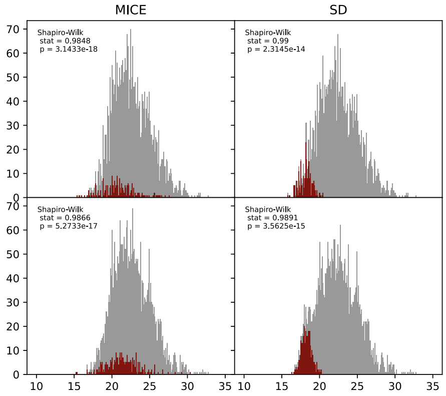

Figure S7: Imputation graphs example. The grey distribution represents the observed (quantified) peptide intensities, while the red distribution represents the imputed values generated using MICE on the left and Down-shifted Gaussian on the right to account for missing data.

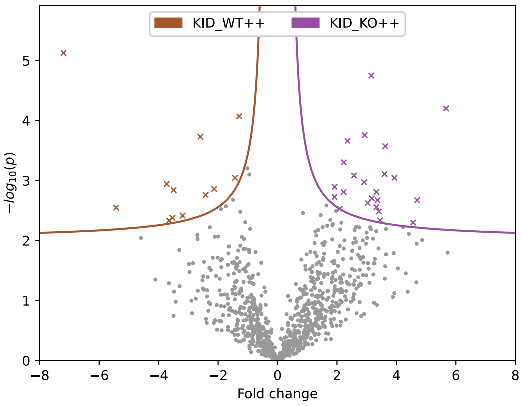

Figure S8: Volcano plot example. Upregulated proteins are shown in purple and downregulated proteins are shown in orange.

$$f_M = -log_2\left(\frac{1}{P}\sum_{n=1}^{P}\sum_{i=1}^{N}\sum_{j=i}^{N}\left(\left(j - i + \left\lceil\frac{N}{12}\right\rceil\right) \times \left(\overline{I_{n,i,e_1}} - \overline{I_{n,i,e_2}}\right) \times \left(\overline{I_{n,j,e_1}} - \overline{I_{n,j,e_2}}\right)\right)\right)$$

N–Number of fractions per condition

P–Number of all peptides of a specific protein

$\overline{I_{p,f,e}}$–Normalized average intensity of peptide p in fraction f and condition e

Equation S1: Mismatch factor equation. The normalization type is set by the user and can be either group-dependent or group-independent.
